# Prediction of Recurrence Risk in Solid Pseudopapillary Neoplasm of the Pancreas: Single-Institution Experience

**DOI:** 10.7759/cureus.17541

**Published:** 2021-08-29

**Authors:** Jalaj Rathi, Gajendra Anuragi, Livin Jose J R, Prabhakaran R, Sugumar C, Naganath Babu O L

**Affiliations:** 1 Surgical Gastroenterology, Madras Medical College, Chennai, IND

**Keywords:** solid pseudopapillary neoplasm, pancreas, recurrence, risk factors, prediction

## Abstract

Background: Solid pseudopapillary neoplasm (SPN) of the pancreas is a low-grade malignant neoplasm with unpredictable behavior. Factors associated with recurrence were not conclusively identified. The aim of this study is to define the clinicopathological criteria for recurrence risk prediction in SPNs based on the most recent scientific evidence and to present our experience with SPNs.

Methods: A retrospective review of patients with SPNs operated on in our institution from June 2012 to June 2018 was completed. Patient characteristics and clinical outcomes were analyzed. A detailed literature review was performed to evaluate the factors associated with the recurrence of SPNs.

Results: The cohort consisted of 13 female patients with a median age of 24 years and a mean tumor size of 7.7 cm. Body and tail (53.8%) were the most common location, and distal pancreatectomy with splenectomy was the prevalent surgical procedure. One patient of SPN operated on for local recurrence after 11 years which had high-grade malignant histological features on the previously resected tumor. At a median follow-up of 42 months (range 36 to 108), all patients were disease free and alive. The proposed criteria for predicting recurrence in SPNs include tumor size >8 cm, synchronous metastasis, malignant SPN (according to 2000 or 2010 World Health Organization [WHO] criteria), lymphovascular invasion, pancreatic parenchymal invasion, and high Ki-67 index (>4%). All these are worse prognostic factors and should be considered as high-risk factors for postoperative relapse.

Conclusion: The above-mentioned criteria can better predict SPN recurrence. Patients with high-risk features should undergo an extended follow-up.

## Introduction

The solid pseudopapillary neoplasm (SPN) of the pancreas is classified as a low-grade malignant epithelial neoplasm of uncertain cellular origin in the 2019 World Health Organization (WHO) classification of tumors of the pancreas [1]. SPNs are relatively rare tumors, accounting for approximately 3% of all cases of cystic tumors of the pancreas in the west, while reported as the most common cystic neoplasm of the pancreas (31.7%) in China. The higher prevalence of SPN in the Asian population in the literature supports ethnic predilection of this tumor [2-5]. Recently, there has been steadily increasing recognition and diagnosis of these tumors, probably due to the more frequent use of cross-sectional imaging [6]. The pathogenesis is still unknown. Pancreatic SPNs are characterized by activation of the WNT signaling pathway through the oncogenic mutation in the beta-catenin gene, which is detected in almost all cases [7].

Most SPNs occur in young female patients and are usually solitary, large, well-circumscribed tumors frequently located in the distal part of the pancreas. Surgery remains the mainstay of treatment [8]. On histopathological analysis, they are composed of a mixture of solid and cystic patterns, blood lakes, and pseudopapillae within a fibrous capsule. Tumor cells of SPN are characteristically positive for vimentin, CD10, CD56, and α-1-antitrypsin [9-11]. It has low malignant potential, and following complete resection has an excellent long-term prognosis. However, despite the low malignancy, up to 10% of the patients experience recurrence or distant metastasis after surgical resection [12]. The exact biological behavior of SPNs is still unknown, and histological features have not been found to correlate with the prognosis of these tumors. Although factors associated with the relapses of SPNs have been reported in several studies, no conclusive criteria have been established till now for predicting the recurrence of SPNs [13-25].

The main objective of this study was to identify the clinical and pathological factors that can predict the risk of recurrence after surgical resection of SPNs based on the current scientific evidence. This will help in determining prognosis and follow-up strategies for the subset of patients who will require long-term monitoring. We present our experience with 13 pancreatic SPN cases in this report.

## Materials and methods

Case series

A retrospective review of patients was performed from June 2012 to June 2018 at the Rajiv Gandhi Government General Hospital, a high-volume tertiary care center in Chennai. We obtained the prospectively maintained databases of patients who underwent surgical resection for cystic lesions of the pancreas. Pathologically confirmed SPN cases were selected, and clinicopathological characteristics including age, sex, tumor size, stage, type of surgery, perioperative findings, histological features, and follow-up information were collected and analyzed. Disease status was assessed at the time of the last follow-up. Data were expressed as median (range) or mean ± standard deviation and frequencies (percentages), depending on data distribution and type.

Literature review

A literature search was performed on the PubMed database for relevant articles from the inception to April 2021 using the keywords ‘Solid pseudopapillary’, ‘tumor’, OR ‘neoplasm’ combined with other terms such as ‘Pancreas’, ‘Recurrence’, ‘Prediction’, ‘Risk factors’, ‘Prognosis’, and NOT Report [Title/Abstract].

Only full-text articles published in the English language were included. Studies that investigated the recurrences of SPNs after surgical resection and focused on the association between the risk factors and recurrence were enrolled. The analysis was limited to studies having a cohort of ≥30 cases with a minimum of three-year follow-up. Literature reviews and studies lacking sufficient data to investigate SPN recurrence were excluded. In case of duplicate publications with overlapping patients, the largest and recent series were included.

Thirteen studies were chosen for final analysis. Data were extracted from each study, and relevant data were compiled and summarized. The following risk factors that are potentially related to SPN recurrence were analyzed: gender, tumor size, location, stage IV disease, resection margins, lymph node status, WHO criteria for malignancy, lymphovascular invasion (LVI), perineural invasion (PNI), capsular invasion (CI), pancreatic parenchymal invasion (PPI), and Ki-67 index.

Factors that demonstrated a univariate association with recurrence at a significance level of P<0.05 were identified and marked as positive (P), while characteristics with no association were indicated with negative (N). When a factor was positive in three or more studies, it was designated as a recurrence risk predictor and included in the criteria to predict the prognosis of SPN.

## Results

Case series: patient characteristics and outcomes

The clinicopathological characteristics of 13 patients who underwent resection in our institute are listed in Table 1. All patients were female with a median age of 24 years (range 15 to 73 years). The most common symptom was abdominal pain and discomfort (76.9%), while 23.1% of patients were incidentally diagnosed. The mean diameter of tumors was 7.7 cm (±2.7). Tumors were located in the pancreatic head in two patients (15.4%), combined body and tail in seven patients (53.8%), and tail region in four patients (30.8%). Only one patient had elevated serum carbohydrate antigen 19-9 (CA 19-9) levels.

**Table 1:** Clinicopathological characteristics of resected pancreatic SPNs in our case series (n = 13) CA 19-9: carbohydrate antigen 19-9, DPS: distal pancreatectomy with splenectomy, PD: pancreaticoduodenectomy, SPN: solid pseudopapillary neoplasm.

Clinical Features	Frequency (%)
Median age, year	24 (15-73)
Sex	
Male	0 (0)
Female	13 (100)
Symptoms	
Abdominal discomfort/pain	10 (76.9)
Palpable mass	2 (15.4)
Weight loss	4 (30.8)
Asymptomatic	3 (23.1)
Tumor location	
Head	2 (15.4)
Body + tail	7 (53.8)
Tail	4 (30.8)
Mean tumor size, cm	7.7 ± 2.7
CA 19-9	
Normal	12 (92.3)
Elevated	1 (7.7)
Recurrent tumor	1 (7.7)
Recurrence pattern	Local
Time to recurrence, months	134
Surgical procedure	
Enucleation	1 (7.7)
DPS	9 (69.2)
Spleen preserving distal pancreatectomy	1 (7.7)
PD	2 (15.4)
Surgery type	
Open surgery	11 (84.6)
Minimally invasive	2 (15.4)
R0 resection	13 (100)
Microscopic features	
Benign features	12 (92.3)
High-grade carcinoma	1 (7.7)
Stage IV disease	0 (0)
Median follow-up, months	42 (36-108)

The most common surgical procedure done was distal pancreatectomy with splenectomy (DPS) in nine patients (69.2%), one patient (7.7%) underwent spleen preserving distal pancreatectomy. Pancreaticoduodenectomy (PD) was done in two patients (15.4%) with portal vein resection, and reconstruction was done in one case. One patient presented with local recurrence of SPN after 11 years, for which enucleation was performed with negative resection margins. This patient had previously undergone a spleen preserving distal pancreatectomy with LVI, PPI, and extensive mitosis on histopathology. R0 resection was achieved in all cases. Five patients (38.5%) had postoperative complications, the most prevalent of which was pancreatic fistula (30.8 %), mainly associated with PD. All of them were biochemical fistulas and successfully managed conservatively. At a median follow-up of 42 months (range 36 to 108), all patients were disease free and alive. None of the patients had tumor recurrence after surgery.

Literature review and analysis

Thirteen articles published between 2008 and 2021 were reviewed [13-25]. All of them were retrospective cohort studies. A total of 1,850 patients ranged from 32 to 375 were included in these studies. A large number of the patients were contributed from South Korea and China containing four studies from each country. The general characteristics of the included studies are presented in Table 2.

**Table 2:** General characteristics of patients with SPN in selected studies *Median. NA: not available, S/L: systemic/local recurrence, SPN: solid pseudopapillary neoplasm.

Author	Year	Country	Total Cases	Female (%)	Mean Age (year)	Mean Size (cm)	Mean Follow-Up (months)	Recurrence (%)	Type S/L	Mean Time for Recurrence (months)
Machado et al. [13]	2008	Brazil	34	79	23*	7	84	2 (5.9)	1/1	NA
Kim et al. [14]	2011	South Korea	114	86.9	36*	4.2*	57*	4 (3.5)	3/1	44 (14–82)
Estrella et al. [15]	2014	USA	64	84	33	5	76*	5 (7.8)	5/0	72 (40–91)
Kang et al. [16]	2014	South Korea	351	90.3	36.8	5.7	NA	9 (2.6)	8/1	18.6* (6.6–81.7)
Kim et al. [17]	2014	South Korea	106	80.2	36*	4.5*	56.9*	2 (1.9)	1/1	NA
Serrano et al. [18]	2014	Canada	32	81.25	35.6	4.7	43*	3 (9.4)	3/0	72 (60–84)
Marchegiani et al. [19]	2016	Italy, USA	131	86.3	33*	4	62*	2 (1.5)	2/0	64 (56–72)
Lubezky et al. [20]	2017	Israel	32	90.6	28.4	5.9	49.2	3 (9.4)	3/0	52 (12-84)
Xu et al. [21]	2017	China	121	76.9	33.7	5	42.7	3 (2.5)	3/0	NA
Liu et al. [22]	2019	China	243	74.5	35.3	4.83	46*	4 (1.6)	3/1	NA
Wu et al. [23]	2019	China	54	83.3	32.7	5.8	60	4 (7.4)	4/0	31.5 (14.4-72)
Lee et al. [24]	2021	South Korea	375	80.3	35.1	4.6	39*	8 (2.1)	4/4	67* (18-170)
Yang et al. [25]	2021	China	193	78.2	35.1	5.2	53*	7 (3.6)	7/0	33* (4-72)

A total of 1,520 (82.2%) patients were female, with a female-to-male ratio of 5.6:1. The mean/median follow-up was ranging from 39 to 84 months. Pooled estimates from studies showed that 56 patients developed recurrence during the follow-up with an overall recurrence rate of 3% (1.5% to 9.4%). The majority (84%) of the patients experienced distant recurrence with or without local recurrence, whereas 16% (9/56) patients developed local recurrence. The most common metastatic site was liver followed by peritoneum. The vast majority of patients survived the recurrence. The time to recurrence was available in nine articles for 45 patients, and the median time to recurrence differed very much among studies between 1.5 years and 6 years [14-16,18-20,23-25]. The shortest recurrence was reported at 4 months and the longest at 170 months.

Risk factors associated with recurrence

The association between SPN recurrence and clinicopathological factors is shown in Table 3. Clinical factors associated with the recurrence were tumor size and stage IV disease at the time of presentation. Seven studies with various size criteria reported that larger tumors increase the risk of recurrences of SPNs [14-17,20,24,25]. Combined results indicate that a tumor size of more than 8 cm represents the optimal cut-off value. Synchronous metastasis was found to increase the risk of relapse in seven studies [14-16,18,21,22,24]. Gender and location did not have any influence on postoperative recurrences.

**Table 3:** Predictors for recurrence risk of SPN after surgical resection P* mentioned only in two of four cases in the study. WHO: World Health Organization, LVI: lymphovascular invasion, PNI: perineural invasion, CI: capsular invasion, PPI: pancreatic parenchymal invasion, SPN: solid pseudopapillary neoplasm, P: positive association, N: no association.

Author/ Year	Sex	Tumor Size	Location	Stage IV	Positive Margin	Lymph Node Spread	WHO Criteria 2000/2010	LVI	PNI	CI	PPI	Ki-67
Machado et al. [13]	N	N						P			P	
Kim et al. [14]		P ≥ 13 cm		P			P		N		N	
Estrella et al. [15]	N	P > 10 cm	N	P	N	P		N	N			
Kang et al. [16]	N	P > 8 cm	N	P	N	P	P/P	N	N	N	N	
Kim et al. [17]	N	P ≥ 5 cm					–/P					
Serrano et al. [18]	N	N		P	N	N		P	N	N	N	
Marchegiani et al. [19]				N	N	N	P	N	N	P	P	
Lubezky et al. [20]		P ≥ 8 cm					N					
Xu et al. [21]	N	N	N	P		N	–/P	P	N		P	
Liu et al. [22]				P								P* 30%
Wu et al. [23]						N	P	P	N		N	P > 4%
Lee et al. [24]	N	P > 4 cm	N	P		N		P	N		N	
Yang et al. [25]	N	P > 10 cm	N					P	N	N	N	P > 20%

Among pathological factors, WHO criteria for malignancy, LVI, PPI, and high Ki-67 index were associated with recurrence risk. According to the 2000 WHO criteria, angioinvasion, perineural invasion, or deep invasion into the surrounding pancreatic parenchyma indicates malignant behavior [26]. However, subsequently in 2010 and 2019 WHO classification, all SPNs were categorized as low-grade malignant neoplasms regardless of microscopic malignant features. In addition, SPNs with extremely aggressive clinical behavior were termed ‘SPN with high-grade carcinoma’ which are characterized by diffuse cellular sheets of tumor cells, increased nuclear atypia, and high mitotic count and are considered a histological subtype [1,27].

Most studies preferred to classify SPNs with malignant potential using the 2000 WHO histological criteria. Four studies found a positive correlation between this criteria and tumor recurrence [14,16,19,23]. In addition, three studies also assessed relapse using 2010 WHO criteria for high-grade malignancy and reported positive association [16,17,21]. So, it was found that both 2000 and 2010 WHO malignancy criteria well predicted the SPNs recurrence after resection.

LVI was found to be significantly associated with increased recurrence in six studies [13,18,21,23-25]. PPI was linked to a higher risk of recurrence in three studies [13,19,21]. High Ki-67 index correlated with SPN relapse in three recently published studies [22,23,25]. However, there was no statistically significant correlation between SPN recurrence and other malignant factors (i.e., perineural invasion, capsular invasion, and lymph node metastasis) in most of the reviewed studies. Interestingly, R1 resection margins investigated in four studies did not show any correlation with the postoperative relapse of SPNs [15,16,18,19].

## Discussion

SPNs are relatively rare neoplasms with indolent behavior and low-grade malignancy potential. Patients with recurrences or metastasis usually have long-term survival after re-resection or metastasectomy. Therefore, it is vital to identify risk factors for tumor recurrence. However, due to the rarity of SPNs, studies on the recurrence risk assessment are limited, and heterogeneity of the results among different studies has failed to produce a consensus in predicting the biological behavior of SPNs. In this review, risk factors for aggressive SPNs associated with high recurrence were identified and defined.

According to the current data, 3% of individuals with SPN experience recurrence after resection. Smaller studies have higher recurrence rates (up to 9.4%), whereas high-volume centers have seen low recurrences (<3%). A similar observation was made in the systematic review conducted by Yepuri et al. which reported an overall recurrence rate of 2% (44/1,897), while Law et al. noted a higher recurrence rate of 4.4% (86/1,866) in their review [6,28]. So far, no patient in our cohort presented with recurrence during follow-up.

Currently, no accurate staging system has been developed to guide the management and follow-up for SPN patients after surgery. Our findings suggest that clinical guidelines for follow-up strategy should be based on tumor size (>8 cm), initial stage IV disease, microscopic malignant characteristics (LVI, PPI, and WHO malignancy criteria), and high Ki-67 index. The patient presented with recurrent SPN in our case series had a 9 cm tumor with LVI, PPI, and extensive mitosis on histology at the time of the first resection, which was consistent with these criteria. The Ki-67 index was not measured in this patient. Two meta-analyses published recently also evaluated clinicopathological factors associated with recurrence. Gao et al. included 10 studies with 1,091 patients; the pooled results suggested that patients with tumor diameter >5 cm, synchronous metastasis, and LVI were prone to suffer from the recurrences of SPNs which is in line with our study. Additionally, lymph node metastasis and positive margin were also found to have a significant risk of recurrence in their study [29]. Yepuri et al. reviewed that 33 studies reporting on 1,897 non-metastatic SPN patients identified male gender, positive lymph nodes, R1 margins, and LVI were associated with a significantly increased risk of recurrence. Stage IV patients were excluded, and tumor size was not analyzed in their review [28].

Synchronous lymph node metastasis is exceptional in SPNs, and positive lymph node was found in only one case in two larger case series by Kang et al. (1/351) and Lee et al. (1/375) [16,24]. This is supported by the fact that lymph node recurrence is also very rare in SPNs after resection. So, routine lymph node dissection is not advised in SPNs but can be considered selectively in high-risk cases.

Although both meta-analyses identified positive margin as a risk factor for relapse, it is worth noting that many other studies have found that patients who had R1 resection had the same clinical results as those who had negative margin [15,16,18,19,30]. Long-term survival is expected even after R1 resection due to the indolent clinical behavior of SPNs. In our analysis, eight studies assessed the effect of gender on tumor aggressiveness and relapse; however, none of them found a link between male gender and tumor recurrence [13,15-18,21,24,25].

Four studies revealed that recurrence was more common in patients with malignant SPN which fulfilled the 2000 WHO criteria in univariate analysis [14,16,19,23]. However, some characteristics according to the 2000 WHO criteria such as capsular invasion and perineural invasion have failed to predict recurrence in multivariate analysis [16,21]. The 2010 WHO criteria for high-grade malignancy may better distinguish recurrence risk groups [16,17,21]. However, there are still no reliable preoperative criteria for the diagnosis of malignant SPNs.

LVI was found to be an independent poor prognostic factor for recurrence in six studies. Lee et al. noted that apart from predicting relapse, LVI was also correlated with other malignant histopathological factors significantly [24]. Yepuri et al. have detected LVI in patients who presented with very late recurrences [28]. Therefore, it is crucial to identify LVI precisely during histopathological analysis as it strongly correlates with the recurrence of SPNs. PPI was the predominant factor in predicting malignant behavior and relapse in three studies [13,19,21]. In addition, one study discovered that PPI was the most frequent pathological feature suggestive of malignancy in SPN [19]. PPI was found to be related to both local and systemic recurrence.

The Ki-67 expression has prognostic value in a variety of malignancies. Yang et al. in 2016 published their experience of 71 patients along with systematic analysis of 163 patients found that Ki-67 index ≥4% was significantly associated with poorer recurrence-free survival (RFS) in SPNs [30]. This study established Ki-67 as a supportive marker to histopathology in predicting tumor relapse. This discovery was later validated by Wu et al. in their study [23]. Subsequently, Yang et al. in a recent multicenter analysis developed a novel grading system ‘The Fudan Prognostic Index’ based on the combination of Ki-67 and tumor size. Multivariate analysis in their study revealed that tumor size >10 cm and Ki-67 >20% were independent predictors for RFS and considered as high-risk factors for recurrence [25].

The majority of the patients in this review developed recurrence within five years; however, recurrence was not uncommon beyond five years. This is in concurrence with the previous report by Yepuri et al. which found that a median recurrence time of 41 months with one-fourth of the patients had recurrence after five years [28]. Similarly, Law et al. noted a mean time to recurrence of 50.5 months [6]. This emphasizes the importance of long-term follow-up in detecting recurrences, especially for those who are at high risk of recurrence.

The present study has some limitations. Relatively few studies investigated the malignant potential and risk factors associated with the recurrence of pancreatic SPNs, which may have influenced our results. The best evidence currently available is based predominantly on single-institution case series and retrospective investigations. In view of these limitations, well-designed multicenter large-scale studies with long-term follow-up are needed to validate the predictive criteria of this study and to determine the optimal surgical strategy and standard follow-up protocol for SPNs.

## Conclusions

Recurrence is experienced in only 3% of patients with SPN after resection, and the majority of them occurred within five years; thus, long-term follow-up is required. Patients with high-risk characteristics such as large tumor size, synchronous metastasis, malignant SPN, LVI, PPI, and high Ki-67 index are associated with recurrence and should undergo an extended follow-up, considering that resection of relapses can still achieve long-term survival. Finally, improved recurrence prediction for SPN patients could modify surveillance strategies, allow for early re-resection, and consider for adjuvant therapy in high-risk groups of patients.
